# A comparison of cryopreservation methods: Slow-cooling vs. rapid-cooling based on cell viability, oxidative stress, apoptosis, and CD34^+ ^enumeration of human umbilical cord blood mononucleated cells

**DOI:** 10.1186/1756-0500-4-371

**Published:** 2011-09-26

**Authors:** Tono Djuwantono, Firman F Wirakusumah, Tri H Achmad, Ferry Sandra, Danny Halim, Ahmad Faried

**Affiliations:** 1 Department of Obstetrics and Gynecology, Faculty of Medicine, Universitas Padjadjaran-Dr. Hasan Sadikin General Hospital, Bandung, Indonesia; 2 Stem Cell Working Group, Faculty of Medicine, Universitas Padjadjaran-Dr. Hasan Sadikin General Hospital, Bandung, Indonesia; 3 Stem Cell and Cancer Institute, Jakarta, Indonesia

**Keywords:** Human umbilical cord blood, hematopoietic stem cell, cryopreservation, slow-cooling, rapid-cooling, cell viability, malondialdehyde, apoptosis, CD34^+^

## Abstract

**Background:**

The finding of human umbilical cord blood as one of the most likely sources of hematopoietic stem cells offers a less invasive alternative for the need of hematopoietic stem cell transplantation. Due to the once-in-a-life time chance of collecting it, an optimum cryopreservation method that can preserve the life and function of the cells contained is critically needed.

**Methods:**

Until now, slow-cooling has been the routine method of cryopreservation; however, rapid-cooling offers a simple, efficient, and harmless method for preserving the life and function of the desired cells. Therefore, this study was conducted to compare the effectiveness of slow- and rapid-cooling to preserve umbilical cord blood of mononucleated cells suspected of containing hematopoietic stem cells. The parameters used in this study were differences in cell viability, malondialdehyde content, and apoptosis level. The identification of hematopoietic stem cells themselves was carried out by enumerating CD34^+ ^in a flow cytometer.

**Results:**

Our results showed that mononucleated cell viability after rapid-cooling (91.9%) was significantly higher than that after slow-cooling (75.5%), with a *p *value = 0.003. Interestingly, the malondialdehyde level in the mononucleated cell population after rapid-cooling (56.45 μM) was also significantly higher than that after slow-cooling (33.25 μM), with a *p *value < 0.001. The apoptosis level in rapid-cooling population (5.18%) was not significantly different from that of the mononucleated cell population that underwent slow-cooling (3.81%), with a *p *value = 0.138. However, CD34^+ ^enumeration was much higher in the population that underwent slow-cooling (23.32 cell/μl) than in the one that underwent rapid-cooling (2.47 cell/μl), with a *p *value = 0.001.

**Conclusions:**

Rapid-cooling is a potential cryopreservation method to be used to preserve the umbilical cord blood of mononucleated cells, although further optimization of the number of CD34^+ ^cells after rapid-cooling is critically needed.

## Background

The success of the first human umbilical cord blood transplantation in 1988 has opened a new perspective on the use of human umbilical cord blood, which is usually discarded after the delivery process. Further research about the properties of human umbilical cord blood led to reports that there are some types of stem cells in umbilical cord blood mononucleated cells, including hematopoietic stem cells. Equal to any other type of stem cells, hematopoietic stem cells have some unique characteristics, such as the ability to self-renew and differentiate into every type of cell in hematopoietic lineages. Until now, at least three sources are known for their potential to become the source of hematopoietic stem cells, i.e., the bone marrow, umbilical cord, and peripheral blood. Of the three, bone marrow is the most commonly used source of hematopoietic stem cells for transplantation. Although it has been proven to be quite effective, the collection of hematopoietic stem cells from bone marrow is an invasive and traumatic procedure. On the other hand, the use of peripheral blood requires a preliminary procedure, such as the administration of the granulocyte colony stimulating factor (G-CSF) prior to collection.

Research by Broxmeyer *et al*. showed that umbilical cord blood is rich in adult stem cells, including hematopoietic stem cells [1]. Because the procedure is less invasive and not traumatic, umbilical cord blood offers a safer and more effective procedure for patients in need of hematopoietic stem cell transplantation. It has also been proven that hematopoietic stem cells obtained from umbilical cord blood have less imunological properties than hematopoietic stem cell obtained from umbilical cord blood have fewer immunological properties than hematopoietic stem cells obtained from any other sources; therefore, their use presents a lower risk of Graft versus Host Disease (GvHD) in allogenic hematopoietic stem cell transplantation. The ratio between hematopoietic stem cells and other mononucleated cells is also considerably high, ranging from 1: 10^4 ^to 1: 10^5 ^cells. Currently, hematopoietic stem cell transplantation is widely used for non-malignant and malignant hematological diseases [2-4].

Because umbilical cord blood can be obtained only once in a person's lifetime, the cryopreservation of umbilical cord blood mononucleated cells is one of the most important steps to ensure the future use of hematopoietic stem cells. The optimum method of cryopreservation should preserve the number and functional quality of hematopoietic stem cells. Currently, slow-cooling is the most frequently used method of cryopreservation. This method has been widely used to preserve various types of cells, tissues, and even organs. Since each type of cell has its own characteristics, researchers believe that each cell should be preserved according to its specific characteristics [5]. Several factors known to contribute to the success rate of cryopreservation are the freezing and thawing rate and the type and amount of cryoprotectant used in the medium [6]. Logically, those factors must be adjusted according to the type of desired cells or tissues.

Slow-cooling requires gradually declining temperature with certain freezing rates. The consequences of the slow and gradual freezing used in the slow-cooling method have been demonstrated to cause cellular injury, resulting in cell death, especially in the range of critical temperatures from -15°C to -60°C. In the context of freezing and thawing, cells must pass through this critical range of temperatures twice [7]. Furthermore, the transition of the preserved cells and surrounding materials from the fluid to the solid state, or vice versa, could cause osmotic injury, resulting in cell death. Due to the possible mechanisms that can cause injury and death in those cells undergoing slow-cooling, rapid-cooling is a potential alternative solution [8].

Rapid-cooling is a cryopreservation method that requires an ultra-rapid cooling rate. This procedure is based on the principle of direct contact between a specimen dissolved in a cryoprotectant medium and liquid nitrogen [9]. Because of its rapid-cooling rate and high viscosity, a specimen that undergoes rapid-cooling is not expected to have any intracellular and extracellular ice crystal formation. To achieve the desired level of viscosity that could turn a fluid into a glassy appearance, a much higher concentration of cryoprotectant than that used for slow-cooling is usually added to the rapid-cooling medium. This step has been one of the most challenging dilemmas because a higher concentration of cryoprotectant would also mean a higher risk of evoking cellular injuries that might lead to cell death. Therefore, research on the optimization of the cooling rates and cryoprotectant concentration should be conducted prior to the routine use of rapid-cooling on any cell types [10,11]. So far, rapid-cooling has been successfully used for the preservation of spermatozoa [11,12], oocytes [13-16], embryos [17-19], and embryonic stem cells [20,21]. This research was conducted due to the need to optimize the cryopreservation method for umbilical cord blood and the weaknesses presented by the slow-cooling method in preserving umbilical cord blood containing hematopoietic stem cells. To evaluate the success of cryopreservation methods, this study used scientific parameters, including cell viability, the formation of malondialdehyde as the product of lipid peroxidation process, the level of apoptosis based on DNA fragmentation, and the enumeration of CD34^+ ^as a marker of a hematopoietic stem cell.

Since there is no obvious optimized cryoprotectant cocktail for rapid-cooling, in particular, for the preservation of hematopoietic stem cells contained in umbilical cord blood, preliminary study was conducted in order to obtain a concentration that could be more effective than that used in the optimized slow-cooling method. The purpose of this study was to scientifically compare the two cryopreservation methods, slow-cooling and rapid-cooling, to determine which one should be used routinely for the preservation of umbilical cord blood in Indonesia.

## Methods

### Sample Collection

Following approval from the Ethical Committee of Hasan Sadikin Hospital and having obtained informed consent from every donor, umbilical cord blood was retrieved from 13 full-term babies at delivery. Other inclusion criteria were: the pregnancy must be a singleton, the fetus must be alive at birth, the mother must be less than 35 years old, and the collected umbilical cord blood must be free of Hepatitis B and HIV infections (clinically tested HBsAg and HIV antibodies). The exclusion criteria used in this study were: a history of smoking and/or alcohol consumption, chronic illness, collection of less than 40 ml of umbilical cord blood, and, lastly, less than 2 × 10^7 ^cells/ml isolated mononucleated cells.

All umbilical cord blood was collected at the Department of Obstetrics and Gynecology of Dr. Hasan Sadikin Hospital, Bandung, Indonesia. First, umbilical cords were clamped and cut immediately after delivery at the nearest point towards the newborn. The injection place was swabbed with 10% povidine iodine or 70% alcohol. The syringe that had been connected to the blood collection bag was then used to collect umbilical cord blood from the previously sterilized injection site [22-24]. The range of blood volume collected using this procedure was between 60 and 150 mL. Umbilical cord blood was kept at room temperature. The blood bags were kept in motion so that the blood would be well mixed with the anticoagulant.

### Isolation of Mononucleated Cells

Mononucleated cells were isolated using Ficoll-Hypaque (density value, 1.077 g/L) on the basis of the principle of differences in gradient density. Briefly, the umbilical cord blood was diluted with phosphate buffer saline human albumin (PBShA) in a ratio of 1: 1. The formulation was then mixed with Ficoll-Hypaque in a ratio of 2: 1. The mixture was centrifugated, which resulted in layers of cells. The thin layer up to the level of the erythrocytes and Ficoll-Hypaque was then gently removed and placed in a tube with PBShA.

### Cryopreservation of Cells Using the Slow-cooling Method

The mononucleated cell suspension was mixed with freezing solutions then incubated for 2 min at room temperature. The mixture was then removed and placed into a 1.8 mL cryovial. Afterwards, the cryovial was put into a canister, which was eventually placed inside the cryo-chamber. The cryo- machine was set at a cooling rate of 2°C/min until it reached -70°C. The cryo- vial was finally moved into a liquid nitrogen tank at a temperature of -196°C.

### Thawing of Samples that Were Previously Cryopreserved Using the Slow-cooling Method

The cryovial was taken out of the liquid nitrogen tank and directly incubated at room temperature for 15 min. The mononucleated cell suspension was then diluted with PBShA at a ratio of 1: 1 and centrifugated at a speed of 300 g for 10 min. The supernatant was then decanted. The pellets were gently removed to be resuspended in 2 ml PBShA. The mixture was centrifugated at a speed of 300 g for 10 min. The remaining pellets were then resuspended in PBShA. Afterwards, the sample was finally ready to be used.

### Cryopreservation of Cells Using the Rapid-cooling

The suspension of mononucleated cells was mixed with a rapid-cooling cocktail at a ratio of 1: 5. The mixture was incubated at room temperature for 1 min. Afterwards, the mixture was removed and placed into 1.8 mL cryovial, and the cryovial was then placed into a canister. The canister was finally placed inside the liquid nitrogen tank at a temperature of -196°C.

### Thawing of the Rapid-cooling Samples

The cryovial was taken out of the liquid nitrogen tank and immediately warmed in a water bath at 37°C for one min. After the suspension became liquefied, cryopreserved mononucleated cells were then diluted in PBShA (1: 1). The mixture was then centrifugated at a speed of 300 g for 10 min. The supernatant was then decanted, and the pellets were resuspended in PBShA. The suspension was centrifugated at 300 g for 10 min. The remaining pellets were suspended in PBShA, and the sample was ready to use.

### Evaluation of Mononucleated Cell Viability

The evaluation of cell viability was conducted using Trypan blue 0.4%. Afterwards, the cells were enumerated using an inverted microscope.

### Evaluation of Lipid Peroxidation

The isolated mononuclear cells at a concentration of 20 × 10^6 ^cells/mL were assessed post-thawing for malondialdehyde as a marker of lipid peroxidation, using the MDA-586 assay kit (MDA-586: Bioxytech™, Portland, OR, USA) according to the manufacturer's instructions. The assay involves a spectrophotometric endpoint at absorbance of 586 nm.

### Evaluation of Apoptosis

The evaluation of apoptosis was carried out with the Terminal deoxynucleotidyl transferase-mediated dUTP Nick End Labeling (TUNEL) assay kit (Roche™, Catalog No: 11684795001) according to the manufacturer's instructions. The results were then analyzed using a flow cytometer (FACSCalibur™).

### CD34^+ ^Cell Enumeration

CD34^+ ^cell labeling was assessed using Anti-CD34 (8B-12)-FITC (BD™ 348053). The whole enumeration process was conducted using a flow cytometer (FACSCalibur™). The analysis of CD34^+ ^enumeration was done using ISHAGE guidelines for CD34^+ ^cell determination [24].

### Statistical Analysis

A normality test using the Saphiro-Wilk test was conducted prior to statistical analysis. To compare the results on the evaluation of cell viability, lipid peroxidation, apoptosis, and enumeration of CD34^+ ^cells, we used the dependent t-test for normally distributed data. On the other hand, for the abnormally distributed data, we used the Wilcoxon signed-rank test. All statistical analyses were conducted using software SPSS^® ^for Windows^® ^13^th ^version. Differences were considered to have statistical significance when the *p *value was ≤ 0.05.

## Results

**Figure 1:**
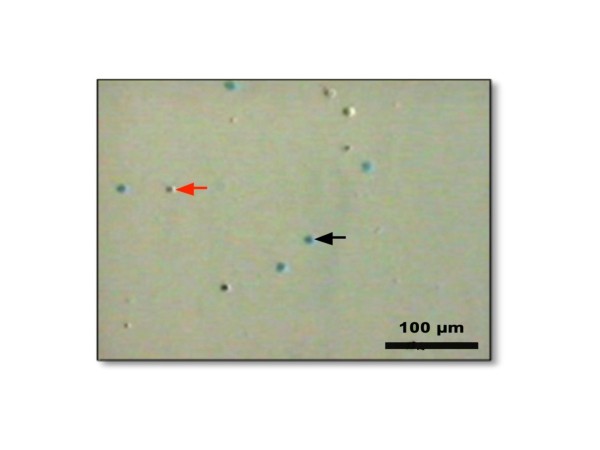
**The viability of mononucleated cells was evaluated with Trypan blue**. The bright round cells (noted by a red arrow) are viable mononucleated cells, whereas the round blue cells (noted by a black arrow) are non-viable mononucleated cells (100× magnification).

This study was conducted over 10 months from January through October, 2009. The total number of donors that were preliminarily evaluated was 23. Based on the inclusion and exclusion criteria, umbilical cord blood from 13 donors was evaluated in this study. The resultant data were the means that were first analyzed using the Saphiro-Wilk test. The results of the evaluation of cell viability after cryopreservation using the slow-cooling and rapid-cooling methods are presented in Table 1. As seen in Table 1, the mean value of cell viability after rapid-cooling was significantly higher than that after slow-cooling. According to the calculation using the Wilcoxon signed-rank test, the level of confidence for these results was 95%, which means that there were significant differences in the mean values of cell viability in the compared sample populations, with a *p *value = 0.003 (*p *value ≤0.05). A microscopic image of cell viability evaluation using Trypan blue is shown in Figure 1.

**Table 1:** The evaluation results of mononucleated cell viability on samples that were cryopreserved by using slow-cooling and rapid-cooling method

Viability	Slow-Cooling	Rapid-Cooling	*p *value*)
(in percentage %)			
Mean (SD)	75.50 (17.53)	91.92 (7.50)	0.003
Median	80.20	94.30	
Range	32.70-93.80	77.80-98.93	

*: *p *value was calculated by using Wilcoxon signed-rank test

**Figure 2:**
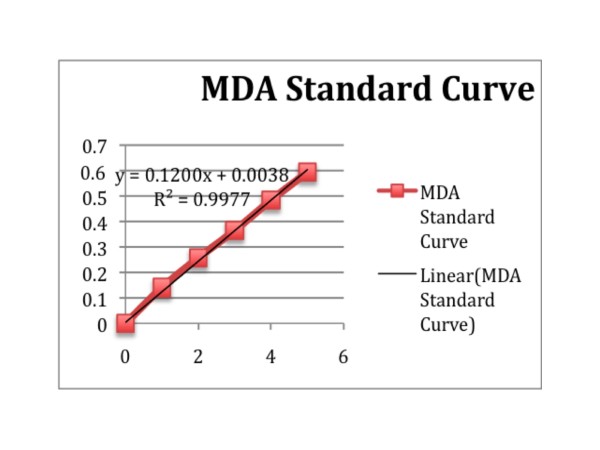
**The MDA standard curve**. This curve was then determined by measuring the absorbance of the cell at 586 nm.

The quality of human umbilical cord blood mononucleated cell populations could also be assessed by the level of oxidative stress. One of the main markers of the ongoing oxidative stress is lipid peroxidation. The major end product of lipid peroxidation is malondialdehyde. The evaluation of malondialdehyde was based on the reaction between N-methyl-2-phenylindole (NMPI) and a malondialdehyde substance. The quantitative value of the absorbance was achieved with a spectrophotometer at 586 nm absorbance [25]. The value of the absorbance from each evaluated sample was then inserted into a formulation provided in the manufacturer's instructions. The variables were defined by the value of the absorbance from the used standards. The malondialdehyde standard curve is shown in Figure 2. Based on Table 2 the value of malondialdehyde in samples that were previously cryopreserved using the rapid-cooling was higher than that in samples previously cryopreserved using the slow-cooling method. According to the calculation using the dependent t-test, the level of confidence for these results was 95%, which means that there were significant differences in the mean values of malondialdehyde between both sample populations, with a *p *value < 0.001 (*p *value ≤0.05). These results mean that there were higher incidences of lipid peroxidation in samples that were previously cryopreserved with the rapid-cooling than in those that were previously cryopreserved with the slow-cooling method. This could also mean that the level of oxidative stress was much higher in the rapid-cooling.

**Table 2:** The evaluation results of malondialdehyde (MDA) in mononucleated cell populations that were cryopreserved by using slow-cooling and rapid-cooling method

Variable	Slow-Cooling	Rapid-Cooling	*p *value*)
MDA (in μM)			
Mean (SD)	33.25 (10.67)	56.45 (9.68)	< 0.001
Median	35.11	57.92	
Range	18.96-51.24	39.27-74.48	

*: *p *value was calculated by using dependent t-test

**Figure 3:**
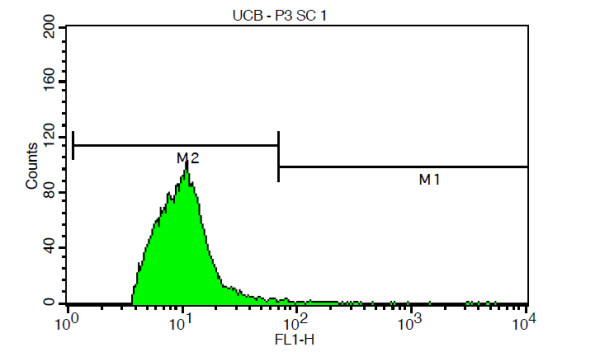
**One result of the TUNEL assay on cells that were previously cryopreserved**. The percentage of cells experiencing DNA fragmentation (M1) was ± 1.15%, while that of cells that did not experience DNA fragmentation was 98.85%.

Apoptosis in umbilical cord blood mononucleated cells that went through the cryopreservation process was also evaluated using the Terminal deoxynucleotidyl transferase-mediated dUTP Nick End Labeling (TUNEL) assay. The results were then analyzed using a flow cytometer [25]. An example taken from one of the results of TUNEL assay is shown in Figure 3. Table 3 clearly shows that the percentage of cells experiencing apoptosis was higher in the samples that were previously cryopreserved using the rapid-cooling than in those that were previously cryopreserved using the slow-cooling method. According to the calculation using the dependent t-test, the level of confidence for this result was 95%, which means that there were no significant differences in the mean values of cells experiencing DNA fragmentation as a sign of apoptosis between both sample populations, with a *p *value < 0.001 (*p *value ≤0.05). Therefore, the differences in the evaluation of apoptosis between two sample populations did not have any significant statistical value.

**Table 3:** The percentage of apoptosis in mononucleated cell populations that were cryopreserved by using slow-cooling and rapid-cooling method

Variable	Slow-Cooling	Rapid-Cooling	*p *value*)
Apoptosis (%)			
Mean (SD)	3.80 (3.06)	5.18 (3.55)	0.138
Median	3.29	4.78	
Range	0.67-9.67	1.20-14.41	

*: *p *value was calculated by using dependent t-test

**Figure 4:**
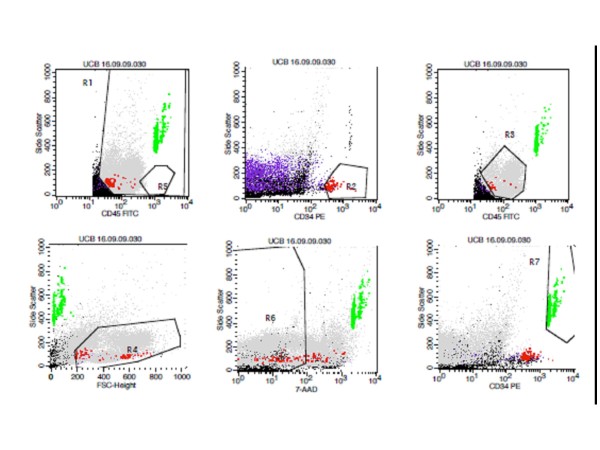
**Analysis of CD34^+ ^enumeration using ISHAGE guidelines**. The results of the CD34^+ ^cell enumeration showed that the number of CD34+ cells in the samples that were previously cryopreserved by the slow-cooling method is 44.99 cell/μL.

The enumeration of CD34^+ ^cells was carried out using a flow cytometer. Analysis of the results was based on the ISHAGE guidelines. The example taken from one of the results obtained with the flow cytometer (Figure 4). Table 4 clearly shows that the number of cells expressing CD34^+ ^was far higher in the sample that was previously cryopreserved using the slow-cooling method than in those that were previously cryopreserved using the rapid-cooling. According to the calculation using the Wilcoxon signed-rank test, the level of confidence for these results was 95%, which means that the number of CD34^+ ^cells in the samples that were previously cryopreserved using the rapid-cooling was significantly lower than that in those that were previously cryopreserved using the slow-cooling method, with a *p *value = 0.001 (*p *value ≤0.05).

**Table 4:** The results of the CD34^+ ^enumeration in mononucleated cell populations that were cryopreserved by using slow-cooling and rapid-cooling method

Variable	Slow-Cooling	Rapid-Cooling	*p *value*)
Number of CD34^+ ^cells (cell/μL)			
Rerata (SD)	23.32 (12.23)	2.47 (3.74)	0.001
Median	22.05	0.90	
Rentang	7.62-53.39	0.11-13.95	

*: *p *value was calculated by using Wilcoxon signed-rank test

## Discussion

It has long been known that the process of cryopreservation and thawing plays an important role in the decline of viability in cryopreserved cells. Factors that are suspected of causing such an occurrence are the type and concentration of the cryoprotectant and the cooling and warming rate used during cryopreservation and thawing. Based on those suspected factors, rapid-cooling was expected to give different outcomes compared to slow-cooling. As stated previously, rapid-cooling was expected to be able to prevent the formation of intracellular and extracellular ice crystal as well as minimize the risk of cell injury that usually occurs within the range of critical temperatures.

As shown in Table 1, our results demonstrate that cell viability after rapid-cooling was significantly higher than that after slow-cooling. The mean value of cell viability after slow-cooling was 75.5%, with overall results ranging from 32.7% to 93.8%. On the other hand, the mean value of cell viability after rapid-cooling was 91.92%, with overall results ranging from 77.80% to 98.93%. Our results were similar to those stated in research by Boyse *et al*., which also showed an overall value of cell viability after cryopreservation with the slow-cooling method ranging from 13.5% to 67.6% [26]. Other results reported by Bayer *et al*. clearly demonstrated that the viability of hematopoietic stem cells after cryopreservation using the conventional slow-cooling method was very low, with a mean value of 62% and an overall value ranging from 51% to 64% [27]. Based on these results, we concluded that rapid-cooling as a cryopreservation method would be better at preserving the viability of umbilical cord blood mononucleated cells. Our conclusion is based on the fact that there was none or less ice crystal formation in the intracellular extracellular region. As reported earlier, there were differences in the type and amount of cryoprotectant used in slow-cooling and rapid-cooling. The media used in the rapid-cooling method in this research were PBShA, dimethyl sulfoxide (DMSO), ethylen glycol (EG), and sucrose (see supplement). These media were different from those used in the slow-cooling method, which consisted of only PBShA, DMSO, and sucrose (see supplement). The addition of ethylene glycol to the cryopreservation medium used in the rapid-cooling method increased the viscosity required by the preserved materials to produce a glass-like appearance or vitreous state. We also suspected that rapid-cooling decreased the potential for injury within the range of critical temperatures. As reported in Methods section, less time was needed for temperature changes within the range of critical temperatures than in the slow-cooling method. The shorter time resulted in a lesser risk of structural lipid changes that could potentially lead to cell death.

It is noteworthy that our results also showed that the malondialdehyde level after rapid-cooling was significantly higher than that after slow-cooling. Malondialdehyde is a substance that has long been known to be a major end product of lipid peroxidation. As shown in Table 2, the mean value of malondialdehyde in cells that were cryopreserved using the rapid-cooling was 56.45 μM, while that of cells that were cryopreserved using the slow-cooling method was 33.25 μM, both with a *p *value of 0.001. The higher value of malondialdehyde shows that there was more lipid peroxidation, resulting in the damage of the cell membrane after rapid-cooling. This result was in obvious contrast to the theory in which rapid-cooling could prevent or minimize damage to the cell membrane due to a very rapid cooling time. Based on the results shown in the evaluation of malondialdehyde, rapid-cooling seems to be a less effective method for the cryopreservation of umbilical cord blood mononucleated cells.

When cell viability is compared, the malondialdehyde value seems highly contradictory. Ideally, less viability would most probably also occur with a higher malondialdehyde value. We assumed that the contradictory value of malondialdehyde and cell viability might be due to the fact that lipid peroxidation is not the only cause of cell death. Many other mechanisms that could lead to necrosis and apoptosis have the potential to cause a decrease in cell viability. Another explanation for this phenomenon is the fact that lipid peroxidation is one of the earliest events in the process of cell death. Therefore, a high value of malondialdehyde could also eventually result in cell death. In the literature studied, we found that this result was similar to those obtained by Schuffner *et al*., in which insignificant changes were reported in lipid peroxidation in samples that had been cryopreserved using the slow-cooling method and previously exposed to a TEST-yolk buffer and glycerol (TYB-G). Furthermore, it has been reported that the most significant change that could lead to cell death in slow-cooling is phosphatidylserine externalization [28].

As shown in Table 3, the value of apoptosis in a population that had been cryopreserved by rapid-cooling was higher than in that exposed to slow-cooling. As reported, the mean percentage of apoptosis in the samples that were cryopreserved using the rapid-cooling samples was 5.18%, with the overall value ranging from 1.20% to 14.41%. On the other hand, the mean percentage of apoptosis in samples that were cryopreserved with the slow-cooling method was 3.80%, with the overall value ranging from 0.67% to 9.67%. The *p *value for both evaluations was 0.138. Hence, we concluded that there were no significant differences between the sample populations. As reported earlier, to evaluate the percentage of cell apoptosis, we used the Terminal deoxynucleotidyl transferase-mediated dUTP Nick End Labeling (TUNEL) assay. The main principle of this essay is based on labeling the the nucleic acid terminal end of the fragmented DNA. DNA fragmentation is a major cause of apoptosis. Unfortunately, DNA fragmentation is not the only event that could lead to apoptosis. Similar results showed by Paasch *et al*. demonstrated that cryopreservation is significantly associated with the activation of caspase-3, caspase-8, and caspase-9, as well as the disruption of the mitochondrial membrane potential, but no significant changes were observed for DNA fragmentation [29]. In contrast, Baumber *et al*. reported that cryopreservation promotes DNA fragmentation [30]. Based on our results, we concluded that both slow-cooling and rapid-cooling have the potential to keep apoptosis at a minimum level in cryopreserved cells. We assumed that DNA fragmentation was not significantly affected by cryopreservation. Therefore, further study, especially regarding the activation of some caspases that could lead to apoptosis, is critically needed.

The most interesting results of our study showed that the enumeration of CD34^+ ^cells was much lower in samples that were cryopreserved by the rapid-cooling method. As shown in Table 4, the mean enumeration value for CD34+ cells in samples that were cryopreserved by slow-cooling was 23.32 cells/μL, with an overall enumeration value ranging from 7.62 cells/μL to 53.39 cells/μL. On the other hand, the mean enumeration value in cells that were cryopreserved by rapid-cooling was 2.47 cells/μL, with an overall enumeration value ranging from 0.11 cells/μL to 13.95 cells/μL. The *p *value for both enumerations was 0.001. Based on these results, we concluded that slow-cooling is far better for preserving the expression of CD34^+ ^cells.

The massive loss in CD34^+ ^cell numbers, especially in samples that were cryopreserved using the rapid-cooling, could be due to several factors. The first is the death of hematopoietic stem cells in populations that were cryopreserved by rapid-cooling. This possibility is doubtful, since the cell viability in samples that were cryopreserved using the rapid-cooling was significantly higher. The second possibility is the change in the cell surface molecules expressed by cells that were once hematopoietic stem cells. This might have happened because of the differentiation into a more mature type of hematopoietic cell or dedifferentiation into more immature hematopoietic stem cells. As reported by Krause *et al*., CD34^+ ^could be used as a single molecular marker of hematopoietic stem cells. It disappeared after the differentiation process [31]. In another publication, Huss reported that more immature hematopoietic stem cells were observed as a result of the negative expression of CD34^+ ^on its surface [32]. Until now, no further explanation concerning a process of cryopreservation that could initiate differentiation and/or dedifferentiation is available. Therefore, more study needs to be conducted to define the specific biomolecular mechanism that effects the differentiation and dedifferentiation process. Another possible explanation for this phenomenon is the damage of the cell surface marker CD34^+ ^because of the direct effect of the cryoprotectant used in rapid-cooling. As reported previously, slow-cooling uses a less cryoprotectant concentration than that required for rapid-cooling. This could result in a decreased risk for extreme dehydration in the cells, which could have an effect on the protein and lipid stability within the membrane. Therefore the protein in the membrane could prevent any denaturation process. On the contrary, a much higher cryoprotectant concentration than that used in the rapid-cooling medium could cause extreme dehydration, which could obviously lead to protein denaturation. Even though rapid-cooling requires a large amount of cryoprotectant, the toxicity is somehow minimized by the very rapid cooling rate in the cryopreservation and thawing process. Unfortunately, the container used was a 1.8 mL cryovial. Containers of this size were chosen because the number of mononucleated cells in every sample reached 3.6 × 10^7 ^cells. Initially, cell concentration in cryopreservation medium was suggested not to be over 2 × 10^7 ^cells/mL. Hence, the most suitable container to be used was a 1.8 mL cryovial. The oversized collector might have resulted in unequal contact of the preserved cells with cold and warm temperatures. This could have caused some cells in the population to be affected by critical temperatures for a longer time than cells that were nearer to the container's wall. For this reason, we concluded that research to determine which containers are best for rapid-cooling is urgently needed. Finally, it is possible that the results of CD34^+ ^enumeration were simply due to unexplainable and undetected CD34^+ ^expression. Therefore, we suggest further study on the functional evaluation of umbilical cord blood mononucleated cell populations after cryopreservation, especially with the use of the rapid-cooling method.

## Conclusions

In summary, rapid-cooling is a potential cryopreservation method for the preservation of umbilical cord blood mononucleated cells, although further optimization of the number of CD34^+ ^cells after rapid-cooling is urgently needed.

## Conflict of interest

The authors declare that they have no competing interests.

## Authors' contributions

TJ, DH, AF carried out the collecting sample and in vitro studies, participated in the molecular assessment and drafted the manuscript. TJ, FS and AF participated in the design of the study and performed the statistical analysis. FFW and THA conceived of the study, and participated in its design and coordination and helped to draft the manuscript. All authors read and approved the final manuscript.

## Supplement

1a The media used in **the rapid-cooling method**:

▸ 2% phosphate buffer saline human albumin (PBShA)

▸ 10% dimethyl sulfoxide (DMSO)

▸ 10% ethylen glycol (EG)

▸ 0.5 M Sukrosa

2a The thawing-media used in **the rapid-cooling method**:

▸ 2% PBShA

▸ 0.5 M Sukrosa

1b The media used in **the slow-cooling method**:

▸ 2% PBShA

▸ 10% DMSO

▸ 0.1 M Sukrosa

2b The thawing-media used in **the slow-cooling method**:

▸ 2% PBShA

▸ 0.2 M Sukrosa
